# Colon cancer and Salicylates

**DOI:** 10.1093/emph/eow009

**Published:** 2016-03-14

**Authors:** Tanveer Dhanoya, John Burn

**Affiliations:** 1 Durham University, Anthropology Department, UK; 2 Clinical Genetics, Newcastle University, Newcastle upon Tyne, UK

## COLON CANCER

Colorectal cancer (CRC), third commonest cancer worldwide, is prevalent in developed countries with highest rates in Europe, North America and Australia and lowest in Asia and Africa; CRC is primarily a disease of modern western lifestyle and is likely evolutionarily novel [1].

## EVOLUTIONARY PERSPECTIVES

Several explanations are supported for increasing rates: we are an aging, sedentary, overweight population compared to hunter-gatherers. Physical activity protects against CRC in both sexes. People are exposed to less UV radiation in the northern latitudes, limiting antineoplastic vitamin D synthesis.

The modern diet lacks fibre and comprises meats, refined carbohydrates and cooked vegetables, prolonging transit time and carcinogen exposure through the long human colon. Eating red meat raises colon cancer risk, possibly by stimulating endogenous insulin secretion, or by producing carcinogens. Conversely, inaccessible carbohydrates stimulate fermentation producing antineoplastic butyrate. An adversely altered microbiome and procarcinogenic bile acid metabolites have long been considered a consequence of the modern diet.

## SALICYLATES

A plant-matter rich diet contains salicylates produced by plants in response to pathogen attack and stress. Sodium salicylate induces apoptosis in cancer cells [2]. Regular ingestion of aspirin, the more palatable acetylsalicylic acid, can prevent cancer: observational studies, randomized polyp prevention studies, long-term cancer follow-up in cardiovascular aspirin trials and randomized trials with cancer as an endpoint confirm this relationship [3]. The first successful, double-blind randomized, controlled trial of aspirin chemoprevention (Cancer Prevention Programme–CAPP2) was carried out in 1009 carriers of Lynch syndrome (hereditary CRC [3]). Two aspirins taken daily for 2 years reduced CRC by >50% after 5 years. Obese participants were particularly protected [4].

Aspirin’s anti-neoplastic effects are generally attributed to salicylic acid inhibiting prostaglandin G/H-synthase 2 (PGHS2) transcription, preventing PGHS2, (COX2), from converting arachidonic acid to potentially tumour-inducing prostaglandins. Aspirin down regulates Sp1, Sp3 and Sp4 transcription factors, reducing expression of proteins including VEGF, reducing tumour cell growth [5]. Additionally, aspirin may only be beneficial in CRCs characterized by a mutated, rather than wild-type PIK3CA gene explaining the effects of aspirin, independently of non-steroidal anti-inflammatory (NSAID) use [6]. Approximately 5000 nmol/ml salicylate inhibits transcription by 50%, although even 100 nmol/ml has an effect. Median serum concentration in vegetarians not taking aspirin drugs is 107 nmol/ml; showing that diet can elevate serum salicylate levels enough to decrease COX2. Assuming salicylic acid is the antineoplastic component in aspirin, dietary intake might be therapeutic [7]. Inhibition of COX2 cannot be the only mechanism by which aspirin prevents cancers, because the anti-neoplastic effect of NSAID drugs does not vary in direct relation to COX2 inhibition. Salicylates trigger cytoplasmic shrinkage and DNA degradation in infected plant cells, a process similar to mammalian apoptosis. Salicylates have also been shown to partially depolarize mitochondria and so inhibit the uptake of calcium ions to mitochondria. Cell proliferation depends on mitochondrial calcium ion uptake and so salicylate inhibition of this uptake may inhibit tumour cell proliferation.

Before modern agriculture, humans would have eaten plants containing higher levels of salicylates, probably sufficient to influence apoptosis, immune response, proliferation and inflammatory response. Salicylates may be considered a lost essential nutrient [2,7].

## FUTURE IMPLICATIONS

Low dose aspirin could prevent CRC. General population use for at least 5 years between the ages of 50–64 would reduce overall mortality despite the risk of gastrointestinal bleeds at a rate of 0.2% per annum [8]. Organic vegetable soups contained significantly higher salicylic acid content [7]. This implies that if food producers restored dietary salicylates by simulating wild stimuli e.g. transient pathogen exposure, and avoidance of herbicides, ingestion of organic food could provide therapeutic serum salicylate levels, though a formal trial is impractical.

**Conflict of interest**: None declared.
